# Immunogenicity and efficacy of pembrolizumab and doxorubicin in a phase I trial for patients with metastatic triple-negative breast cancer

**DOI:** 10.1007/s00262-023-03470-y

**Published:** 2023-06-09

**Authors:** Colt A. Egelston, Weihua Guo, Susan E. Yost, Xuan Ge, Jin Sun Lee, Paul H. Frankel, Yujie Cui, Christopher Ruel, Daniel Schmolze, Mireya Murga, Aileen Tang, Norma Martinez, Misagh Karimi, George Somlo, Peter P. Lee, James R. Waisman, Yuan Yuan

**Affiliations:** 1 Department of Immuno-Oncology, City of Hope Comprehensive Cancer Center, Duarte, CA USA; 2 Department of Medical Oncology & Therapeutics Research, City of Hope Comprehensive Cancer Center, Duarte, CA USA; 3 Department of Statistics, City of Hope Comprehensive Cancer Center, Duarte, CA USA; 4 Department of Pathology, City of Hope Comprehensive Cancer Center, Duarte, CA USA; 5 Division of Medical Oncology, Cedars-Sinai Cancer, Cedars-Sinai Medical Center, 127 S San Vincente Blvd. 7th Floor Los, Angeles, CA 90048 USA

**Keywords:** Pembrolizumab, Doxorubicin, Triple-negative breast cancer

## Abstract

Currently there is a limited understanding for the optimal combination of immune checkpoint inhibitor and chemotherapy for patients with metastatic triple-negative breast cancer (mTNBC). Here we evaluate the safety, efficacy, and immunogenicity of a phase I trial for patients with mTNBC treated with pembrolizumab plus doxorubicin. Patients without prior anthracycline use and 0–2 lines of prior systemic chemotherapies received pembrolizumab and doxorubicin every 3 weeks for 6 cycles followed by pembrolizumab maintenance until disease progression or intolerance. The primary objectives were safety and objective response rate per RECIST 1.1. Best responses included one complete response (CR), five partial responses (PR), two stable disease (SD), and one progression of disease (PD). Overall response rate was 67% (95% CI 13.7%, 78.8%) and clinical benefit rate at 6 months was 56% (95% CI 21.2%, 86.3%). Median PFS was 5.2 months (95% CI 4.7, NA); median OS was 15.6 months (95% CI 13.3, NA). Grade 3–4 AEs per CTCAE 4.0 were neutropenia *n* = 4/10 (40%), leukopenia *n* = 2/10 (20%), lymphopenia *n* = 2/10 (20%), fatigue *n* = 2/10 (20%), and oral mucositis *n* = 1/10 (10%). Immune correlates showed increased frequencies of circulating CD3 + T cells (*p* = 0.03) from pre-treatment to cycle 2 day 1 (C2D1). An expansion of a proliferative exhausted-like PD-1 + CD8 + T cell population was identified in 8/9 patients, and exhausted CD8 + T cells were significantly expanded from pre-treatment to C2D1 in the patient with CR (*p* = 0.01). In summary, anthracycline-naïve patients with mTNBC treated with the combination of pembrolizumab and doxorubicin showed an encouraging response rate and robust T cell response dynamics.

**Trial registration:** NCT02648477.

**Supplementary Information:**

The online version contains supplementary material available at 10.1007/s00262-023-03470-y.

## Introduction

Triple-negative breast cancer (TNBC) accounts for 10–15% of all breast cancers and is characterized by lack of estrogen receptor (ER), progression receptor (PR), and human epidermal growth factor receptor 2 (HER2) overexpression. TNBC is molecularly heterogeneous, and metastatic TNBC (mTNBC) carries poor prognosis due to the overall lack of effective targeted therapy [1]. Recent US Food and Drug Administration (FDA) approvals in TNBC, namely PARP inhibitors for BRCA germline-mutated tumors [2, 3], immune checkpoint inhibitors (ICIs) for programmed death-ligand 1 (PD-L1) positive TNBC [4], and antibody drug conjugate targeting the Trop-2 receptor [5] have changed the landscape of mTNBC treatment and improved patient survival [6]. The combination of ICIs including atezolizumab and pembrolizumab with chemotherapies such as paclitaxel, nab-paclitaxel or gemcitabine/carboplatin have shown promise in the first-line setting for mTNBC [7, 8]. However, limited data are available for second-line or later ICI combinations and for combinations with other chemotherapy agents. There is an unmet need to further test the combination of chemotherapy and ICI combinations in both first and later line settings for breast cancer (BC) patients.

Pembrolizumab, a monoclonal anti-PD-1 antibody, is approved for the treatment of multiple solid tumors [9]. In the KEYNOTE-355 trial of first-line patients with PD-L1 positive (PD-L1 +) TNBC defined by a combined positive score (CPS) ≥ 10 using 22C3 antibody, pembrolizumab plus chemotherapy had improved PFS compared with chemotherapy alone (9.7 vs. 5.6 months; HR 0.65, 95% confidence interval (CI): 0.49–0.86) [8], which led to FDA approval in 2021. The synergetic effects were observed regardless of the chemotherapy backbone that was used: paclitaxel, nab-paclitaxel, or gemcitabine/carboplatin. Other chemotherapy agents, such as eribulin in combination with pembrolizumab, were tested in a phase I/II trial (ENHANCE1) which showed encouraging antitumor activity in PD-L1 + mTNBC, with overall response rate (ORR) of 34.5% in first-line patients and 24% in second-line or later patients, respectively [10]. Anthracycline is one of the main chemotherapy agents that has been used primarily in the neoadjuvant/adjuvant settings for early stage TNBC, and there have been limited studies on its immune modulatory effects. Studies potentially pointing to lesser need of anthracyclines in the adjuvant/neoadjuvant setting have been performed; hence, a larger proportion of BC patients who are anthracycline-naïve may benefit from receiving anthracycline-based therapies in the metastatic setting [11, 12].

Multiple preclinical studies have demonstrated an immune potentiating effect of anthracycline [13]. In a colon cancer mouse model, doxorubicin induced immunogenic cell death (ICD) and elicited a dendritic cell-mediated tumor-specific CD8^+^ T cell response [14]. In addition, in a breast cancer mouse model, doxorubicin selectively depleted myeloid-derived suppressor cells (MDSC) from the tumor microenvironment (TME) [15]. Mattarollo et al. have shown the effect of doxorubicin treatment is dependent on CD8 + T cells and gamma interferon, and doxorubicin treatment enhances tumor antigen-specific proliferation of CD8 + T cells in tumor-draining lymph nodes and promotes tumor infiltration of activated IFN-γ-producing cells [16].

We hypothesized that the combination of pembrolizumab and doxorubicin is synergistic in facilitating both cellular immune response and chemotherapy effects in treatment of mTNBC. The current trial, although small, was designed to test the safety and efficacy of the combination of pembrolizumab and doxorubicin in anthracycline-naïve patients with mTNBC. In addition to the safety and efficacy data, we also report tumor immune biomarkers and peripheral blood immune subset composition including dynamic changes over time that should be validated in larger studies.

## Methods

### Study design and patient population

This open-label single institutional phase I trial for patients with metastatic TNBC was conducted between March 2016 and November 2019 with institutional review board (IRB) approval in accordance with the World Medical Association Declaration of Helsinki, International Conference on Harmonization Good Clinical Practice guidelines, and the US code of federal regulations. Informed voluntary consent forms were signed by all patients prior to study entry. This study is registered at the ClinicalTrials.gov under number NCT02648477. Main eligibility criteria included patients who were 18 years or older with mTNBC defined by ASCO/CAP guideline, no prior anthracycline exposure, measurable disease based on RECIST 1.1, and ≤ 2 prior systemic anticancer therapies in the metastatic setting. Additional inclusion criteria were an Eastern Cooperative Oncology Group (ECOG) performance status 0–1; life expectancy ≥ 3 months; and adequate bone marrow, renal, and hepatic function. Main exclusion criteria included prior anthracycline therapy; prior pembrolizumab therapy; or prior diagnosis of immunodeficiency, use of systemic steroid, or any other form of immunosuppressive therapy within 7 days prior to the first dose of trial treatment.

### Study procedure

Eligible patients received pembrolizumab 200 mg IV with doxorubicin 50–60 mg/m^2^ on day 1 of each 3 weeks cycle. Doxorubicin was started at 50 mg/m^2^ and then escalated to 60 mg/m^2^ based on acceptable toxicity during safety lead in for a total of 6 cycles. After 6 cycles of doxorubicin and pembrolizumab, patients were continued on pembrolizumab maintenance for up to 24 cycles. Response assessments by CT scans were performed at baseline and then every 9 weeks for RECIST 1.1 reading. Patients with complete response (CR) or partial response (PR) were expected to be confirmed by a second examination performed ≥ 4 weeks after the first observation of response. Best overall response of stable disease (SD) required ≥ 1 post-treatment assessment that met the SD criteria > 8 weeks after the start of treatment.

### Clinical response statistics

The primary objective of the study was to evaluate ORR of pembrolizumab plus doxorubicin. The secondary objective was to assess clinical benefit rate (CBR) (no progression for > 24 weeks), progression-free survival (PFS), and overall survival (OS). Additional secondary endpoints were to assess the safety and tolerability of anthracycline plus pembrolizumab regimen. Responses were assessed by RECIST 1.1, and safety analysis was carried out based on toxicities assessed by CTCAE 4.0.

A safety lead-in employing a 3-at-risk rolling design was used [17]. For each treatment, only 3 patients were permitted to be at risk for first cycle toxicities at any one time during the safety-lead-in. Patients needed to be doxorubicin naïve. As a result of this patient selection, we set a discouraging response rate at 15% and an encouraging rate at 34%. The null hypothesis was H0: ORR ≤ 15%, and the alternative was H1: ORR ≥ 34%. Simon’s MinMax two-stage design with a type I error of 10% and a power of 90% was followed. In the case of early stopping, evaluation of patient subsets (e.g., immune phenotype), in consultation with the PI, statistician, sponsor and DSMB, allowed the study to continue for specific subsets following an amendment if it was deemed to be inadequately evaluated and there appeared to be sufficient promise for that subset. If early stopping did not occur, accrual would continue to a total of 36 patients. With 36 patients, 9 patients with an ORR (25%) were required to deem this combination worthy of further evaluation. This maintained the type I error at 10% to reject the null hypothesis and the power at 90% to declare a positive finding if the alternative hypothesis holds. Clinical outcomes including PFS and OS were calculated by the Kaplan–Meier method, and median follow-up was calculated among alive patients. The Clopper–Pearson method was used to calculate 95% CIs for ORR and CBR.

### Tumor immune biomarkers

Tumor biopsies were formalin-fixed paraffin-embedded (FFPE). Percentage of stromal TILs (sTILs) in tumor was evaluated using hematoxylin and eosin (H&E) diagnostic sections per International Immuno-Oncology Biomarker Working Group on Breast Cancer Guidelines [18]. PD-L1 was determined by QualTek Molecular Laboratory (Goleta, CA) using immunohistochemistry (IHC) with 22C3 antibody (Merck & Co, Kenilworth, NJ). PD-L1 was positive if membrane staining was present in at least 1% of cells, or there was a band of PD-L1-positive mononuclear cells at the interface between tumor cells and adjacent stroma. Both tumor and mononuclear cells located adjacent to tumor cells were scored [19, 20].

### Peripheral blood immune correlatives

Peripheral blood was collected at baseline (pre-treatment), C2D1, and post-cycle 3 (C4D1 or C6D1) for flow cytometry analysis. Peripheral blood was obtained using heparin collection tubes, and peripheral blood mononuclear cells (PBMCs) were isolated within 6 h using Ficoll-Paque Separation according to manufacturer’s instructions (GE Healthcare). PBMCs were cryopreserved in 10% DMSO, 90% FBS and thawed rapidly for flow cytometry analysis. Single-cell suspensions were prepared on ice in 2% FBS in PBS. Antibody cocktails were diluted in Brilliant Violet Buffer (BD Biosciences). Samples were acquired using a Cytek Aurora spectral cytometer with user settings established by Spectroflo QC beads. Unmixing and compensation were performed in Spectroflo software using a mix of reference controls from either single stained PBMCs or single stained OneComp compensation beads (eBioscience). Samples were stained with fluorescently tagged antibodies (Supplemental Table 4). Antibodies were titrated for optimal signal-to-noise ratio prior to use. Flow cytometry analysis was performed using Flowjo vX, and the CATALYST R package was used for FlowSOM analysis and UMAP projections [21]. All samples were gated on live, single cells.

### Correlative studies statistics

Graphs and statistics were performed using GraphPad Prism 8.4.3. Statistics were generated using unpaired two-tailed Student T tests or multiple comparisons T tests with Dunnet’s or Holm-Sidak corrections as described. Calculated p values are displayed as *p < 0.05; **p < 0.01; ***p < 0.001; ****p < 0.0001. For all graphs, the mean is represented by a line.

## Results

### Patients

Between March 2016 and November 2019, a total of 10 patients were enrolled and treated with doxorubicin and pembrolizumab. The trial was stopped early due to poor accrual because of difficulty in identifying patients who were anthracycline-naive. All 10 patients were included in the safety evaluation. One patient with chronic respiratory disease developed respiratory failure which led to death and was not evaluable for efficacy. Baseline patient characteristics are listed in Table 1. Median age was 62 years (*n* = 10; 41–87 years); *n* = 7/10 (70%) were white, *n* = 2/10 (20%) Asian and *n* = 1/10 (10%) African American. Median line of therapies was 1 (range 0–2). Visceral and bone metastasis were *n* = 3/10 (30%) lung/liver/bone, *n* = 1/10 (10%) lung only, *n* = 1/10 (10%) liver only, and *n* = 2/10 (20%) bone only.

**Table 1:** Baseline patient characteristics (*N* = 10)

Characteristic	
Age (median, range)	62 (41–87)
Race	
White	7 (70%)
Asian	2 (20%)
African American	1 (10%)
ECOG Performance status	
0	2 (20%)
1	8 (80%)
Initial tumor stage	
Stage I	2 (20%)
Stage II	3 (30%)
Stage III	3 (30%)
Stage IV	2 (20%)
Histology grade at diagnosis	
Grade II	2 (10%)
Grade III	6 (70%)
Unknown	2 (20%)
Prior surgery	
Mastectomy	7 (70%)
Lumpectomy	2 (20%)
No surgery	1 (10%)
Prior radiation	3 (30%)
No prior radiation	7 (70%)
Lines of chemotherapy for metastatic disease	
0	
3	3 (30%)
1	2 (20%)
2	2 (20%)
2 +	3 (30%)
Visceral and bone metastasis	
Lung/liver/bone	3 (30%)
Lung only	1 (10%)
Liver only	1 (10%)
Bone only	2 (20%)
Prior chemotherapy (Neo) adjuvant:	4 (40%)
docetaxel + cyclophosphamide Metastatic:	
Carboplatin + paclitaxel	2 (20%)
Capecitabine	4 (40%)
Gemcitabine	1(10%)
Navelbine	1(10%)
Nab-paclitaxel	2 (20%)
Eribulin	1 (10%)

### Treatment

The first 3 patients received doxorubicin at 50 mg/m^2^ without dose-limiting toxicities (DLT), and dose was escalated to 60 mg/m^2^ for the remaining 7 patients of this study. The recommended phase II dose (RP2D) dose for doxorubicin was 60 mg/m^2^. A total of *n* = 8/10 (80%) patients completed 6 cycles of doxorubicin.;*n* = 2/10 (20%) patients had dose delay (1 due to grade 3 neutropenia and 1 due to flu-like symptoms); *n* = 2/10 (20%) patients on the 60 mg/m^2^ dose had dose reduction to 50 mg/m^2^, one for grade 3 fatigue and one for grade 3 oral mucositis.

### Response and survival

**Fig. 1:**
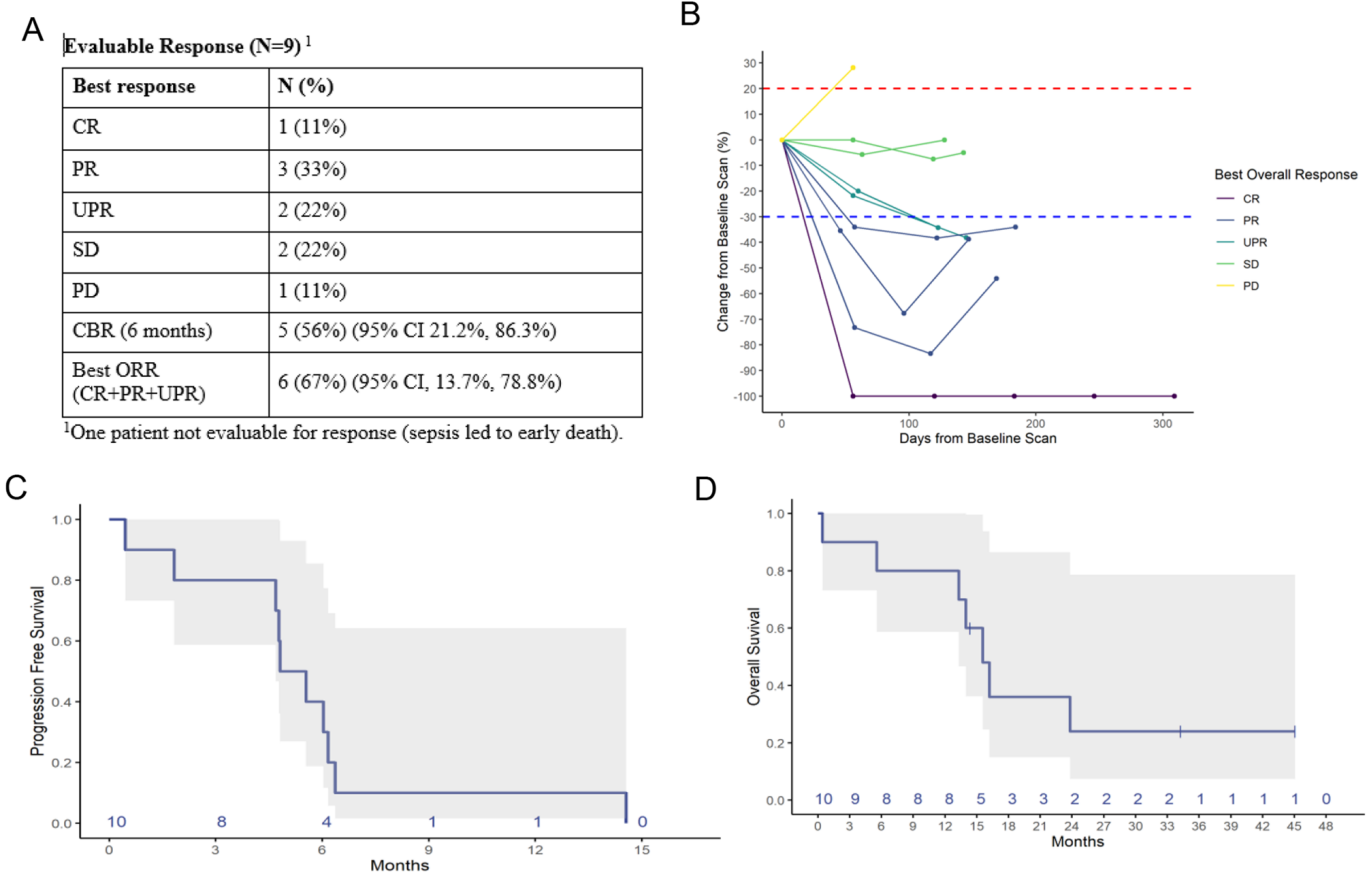
Relative Change in Tumor Size (*n* = 9). **A** Spider plot shows change in tumor from baseline starting at time of protocol therapy with pembrolizumab. One patient had an exceptional response with PFS 14.8 months. **B** Evaluable responses, CBR, and ORR are shown in table. One patient was not evaluable for response (sepsis led to early death); **C** Median PFS was 5.2 months (95% CI 4.7, NA); and D) median OS was 15.6 months (95% CI 13.3, NA)

Of 10 patients treated, one patient (age 87) developed neutropenia, sepsis, and death after 1^st^ dose of therapy, and was not eligible for response assessment. Of 9 evaluable patients, best responses were 1/9 CR (11%), 3/9 PR (33%), 2/9 UPR (22%), 2/9 SD (22%) and 1/9 PD (11%), with a best ORR (CR + PR + UPR) of 6/9 (67%) (95% CI 13.7%, 78.8%). CBR at 6 months was 5/9 (56%) (95% CI 21.2%, 86.3%) (Fig. 1A). The spider plot shows relative changes in tumor size from baseline over time (Fig. 1B). Of the 6/9 responders: one CR patient had a time to first documented response of 56 days, and a duration of response of 388 days (for a total PFS time of 444 days); three PR patients had time to response of first re-staging scan of 46, 47 and 47 days, with a duration of response of 101, 112 and 127 days; and two UPR patients had a time to response of 123 and 194 days, with a duration of response of 23 and 49 days. Median follow-up time was 34.6 months (range 14.5–45.4 months). The median progression-free survival was 5.2 months (95% CI 4.7, NA) (Fig. 1C). The median OS was 15.6 months (95% CI 13.3, NA) (Fig. 1D).

### Exceptional responder

Patient was initially diagnosed with ER/PR-positive, HER2-negative breast cancer in 2002. She was treated with surgery, adjuvant cyclophosphamide methotrexate fluorouracil (CMF), and radiation therapy, followed by 5 years of adjuvant tamoxifen. In 2011, patient who recurred with high-grade TNBC received neoadjuvant docetaxel and cyclophosphamide, followed by bilateral mastectomy. Surgical pathology demonstrated yPT2N1 residual disease. Patient further received adjuvant gemcitabine and cisplatin and then had progressive disease with adenopathy in the axilla and mediastinum. Patient received the following lines of therapy: first-line carboplatin and paclitaxel from September 2015 to December 2016; second-line Xeloda from January 2017 to April 2017; third-line doxorubicin; and 22 cycles pembrolizumab from May 2017 to July 2018. Patient had an exceptional response but progressed on study.

### Treatment associated toxicities

Grade 3–4 adverse events (AEs) per CTCAE 4.0 were neutropenia *n* = 4/10 (40%), leukopenia *n* = 2/10 (20%), lymphopenia *n* = 2/10 (20%), leukemia *n* = 1/10 (10%), fatigue *n* = 2/10 (20%), oral mucositis *n* = 1/10 (10%), and gastroesophageal reflux disease *n* = 1/10 (10%). One patient (age 87) with prior pleural effusion (grade 2) and COPD received one dose of therapy and became neutropenic which led to sepsis and death. Patients experienced multiple AEs including grade 3–4 neutropenia, leukopenia, lymphopenia, hyponatremia, acidosis, alkalosis, hypotension and respiratory failure (*n* = 1/10; 10% each) (Table 2). One patient developed grade 2 hypothyroidism attributed to pembrolizumab, and one patient with prior chemotherapy developed secondary leukemia (ALL) 5 months post-progression. Grade ≥ 3 immune related adverse events (irAEs) attributed by participating investigators were neutropenia *n* = 4/10 (40%), leukopenia *n* = 2/10 (20%), lymphopenia *n* = 2/10 (20%), fatigue *n* = 2/10 (20%), acidosis *n* = 1/10 (10%), alkalosis *n* = 1/10 (10%), dyspnea *n* = 2/10 (20%), and hyponatremia *n* = 1/10 (10%) (Supplemental Table 1).

**Table 2:** Grade 2–4 adverse event with attributions in “definite”, “possible”, “probable” per CTCAE 4.0^1^

Adverse event	Grade 2	Grade 3	Grade 4^1^
All adverse events (worst grade per patient)	5	4	1
Cardiovascular			
Hypotension			1 (10%)^1^
Cardiomegaly	1 (10%)^1^		
Vascular disorders	1 (10%)		
QT prolongation	1 (10%)		
Bone Marrow			
Neutropenia	1 (10%)	3 (30%)	1 (10%)^1^
Leukopenia	4 (40%)	1 (10%)	1 (10%)^1^
Lymphopenia	4 (40%)	1 (10%)	1 (10%)^1^
Leukemia (ALL, 5 months post-progression)			1 (10%)
Anemia	3 (30%)		
T hrombocytopenia	1 (10%)		
Respiratory			
Cough	2 (20%)		
Dyspnea		1 (10%)^1^	
Respiratory failure			1 (10%)^1^
Bronchospasm	1 (10%)		
General			
Hypothyroidism	1 (10%)		
Infusion-related reaction	1 (10%)		
Fatigue	3 (30%)	2 (20%)	
Anorexia	1 (10%)		
Memory impairment	1 (10%)		
Somnolence	1 (10%)		
Alopecia	1 (10%)		
Arthralgia	1 (10%)		
Localized edema	1 (10%)		
Acute kidney injury	1 (10%)		
Gastrointestinal			
Nausea	2 (20%)		
Vomiting	1 (10%)		
Dyspepsia	2 (20%)		
Oral mucositis		1 (10%)	
Gastroesophageal reflux disease (GERD)	1 (10%)	1 (10%)	
Rash maculo-papular	1 (10%)		
Electrolytes			
Hyponatremia		1 (10%)^1^	
Hypokalemia	1 (10%)		
Hypocalcemia	1 (10%)		
Hypophosphatemia	1 (10%)		
Creatinine increased	1 (10%)		
Hypoalbuminemia	1 (10%)		
Acidosis		1 (10%)^1^	
Alkalosis		1 (10%)^1^	
Infection			
Bladder infection	1 (10%)		
Bronchial infection	1 (10%)		
Upper respiratory infection	1 (10%)		
Pain			
Breast pain	1 (10%)		
Back pain	1 (10%)		
Musculoskeletal and connective tissue disorder	1 (10%)		
Neck pain	1 (10%)		

### Tumor immune biomarkers

PD-L1 (22C3) testing showed 4 patients who were PD-L1 positive, 4 patients who were PD-L1 negative, and 2 patients who did not have PD-L1 results. Stromal TILs analysis of available tumor tissue was performed for 4 patients and showed 1 CR patient with 90% TILs (lymph node), 1 UPR patient with 5% TILs (axillary mass), 1 UPR patient with 10% TILs (liver), and 1 PD patient with 3% TILs (axillary mass). Five patients did not have TILs analysis due to exhausted tissue block. Overall, no association between baseline levels of TILs or PD-L1 status with response was observed (Supplemental Table 2). We do note that the patient with CR had 90% TILs in the biopsied lymph node, although we were unable to assess TILs in the patient’s other disease sites such as lung and dermal tissues. It is therefore unclear if the high TIL density is associated with the biopsy site of lymph node or with patient response. Genomic alterations for patients with sequencing results did not show association with response (Supplemental Table 3).

### PMBC immune cell composition

Baseline and on-treatment characteristics of peripheral blood immune cell composition were analyzed in response to therapy. Two high parameter (> 28) spectral cytometry panels were designed to identify both broad immune subsets and detailed T cell subsets. At baseline, we found no association of response and frequencies of CD3 + T cells, natural killer (NK) cells, natural killer T cells (NKT), γδ T cells, or B cells within total CD45 + leukocytes (Supplemental Fig. 1A). Intriguingly, the patient with PD had the highest fraction (11.0% PD vs. 6.3% mean CR/PR/SD) of terminally differentiated NK cells (CD56dim CD16 +) among all NK cells at baseline (Supplemental Fig. 1B). Moreover, the patient with PD also demonstrated fewer naïve B cells (48.4% in PD vs. 75.8% mean in CR/PR/SD and higher levels of plasmablasts (15.2% in PD vs. 1.5% mean in CR/PR/SD) among all B cells compared to other patients (Supplemental Fig. 1C). No differences in the subset composition of monocytes (classical, ClMono; intermediate, IntMono; non-classical, NcMono) or dendritic cells (conventional type 1, cDC1; conventional type 2, cDC2; plasmacytoid, pDC) were observed (Supplemental Fig. 1D and E). We then examined how these major immune populations changed during treatment. From pre-treatment to C2D1, the fraction of CD3 + T cells among total CD45 + cells increased significantly (*p* = 0.008), with no other major changes in general immune subset composition (Supplemental Fig. 1F). Changes in overall conventional T cell frequencies returned to baseline levels post-cycle 3, and a significant reduction in naïve B cells (*p* = 0.02) was observed (Supplemental Fig. 1F).

### T cell compositional changes over the course of treatment

Given the increased frequency of T cells from pre-treatment to C2D1, we next set to dissect features of T cell subsets in greater detail over the course of therapy and in context of tumor response. Within CD8 + and CD4 + T cell populations, we found no significant differences in frequencies of canonical naïve, central memory (CM), effector memory (EM), or effector memory CD45RA + (EMRA) populations at baseline (Supplemental Fig. 2). However, the patient with PD had the lowest frequency of naïve CD8 + T cells (5.7% vs. 22.6% mean in CR/PR/SD) and second lowest frequency of naïve CD4 + T cells (20.1% vs. 39.7% mean in CR/PR/SD) among all patients.

**Fig. 2:**
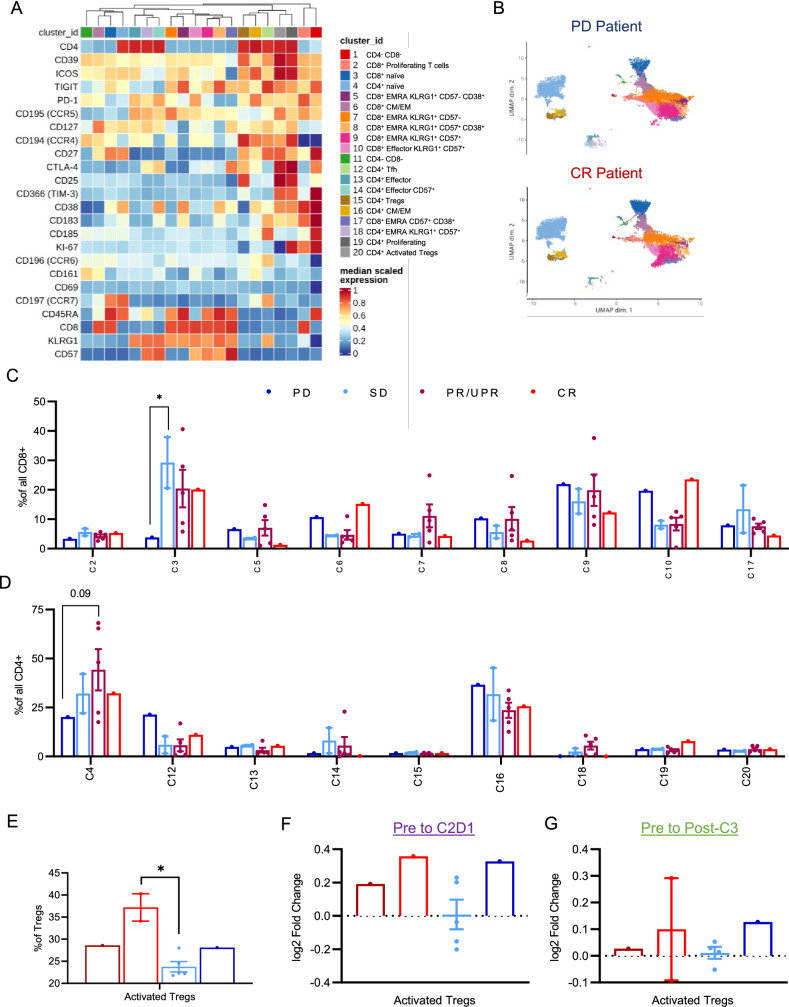
T cell subset in association with response to doxorubicin and pembrolizumab. Circulating T cells were assessed by flow cytometry for complex phenotyping. Dimensionality reduction by the FlowSOM algorithm was performed to identify T cell metaclusters in an unbiased manner. **A** A heatmap of identified clusters displays expression of various surface proteins used for each identified cell cluster. **B** Representative UMAP projections of the PD and CR patients. **C** Percentages of identified T cell clusters as a fraction of total CD8 + T cells or **D** total CD4 + T cells are shown. **E** Baseline percentages of activated (CTLA-4 +) regulatory T cells among total regulatory T cells; **F** fold change of activated Treg percentage from pre-treatment to C2D1; **G** post-cycle 3. *, *p* < 0.05

Unbiased clustering was performed and identified 20 unique clusters of T cell subsets that were then manually annotated based on marker expression (Fig. 2A, B). Two clusters (C1 and C2) lacked CD4 and CD8 expression and were disregarded for the remainder of the analysis. C1 and C2 were naïve CD8 + and CD4 + T cells, respectively, as determined by co-expression of CD45RA and CCR7. This yielded a remaining 9 CD8 + T cell clusters and 9 CD4 + T cell clusters. C6 and C16 were annotated as central memory/effector memory (CM/EM) CD8 + and CD4 + , respectively, based on expression of CD127, CD27, CCR7, and reduced CD45RA expression. C6 and C16 both were composed of cells with varied expression of CCR6 and CCR4, reflecting varied polarization states, and cells expressing CD161, reflecting a quiescent resting memory phenotype. C10 appeared to be a CD8 + effector T cell population expressing both KLRG1 and CD57. C13 and C14 were KLRG1 + effector CD4 + T cells without and with CD57 co-expression, respectively. A number of EMRA CD8 + T cell subsets were observed as clusters C5, C7, C8, C9, and C17 reflecting varied states of senescence (CD57) and activation (CD38). EMRA CD4 + T cells were less heterogenous, CD57 + KLRG1 + , and entirely found in c18. Circulating follicular helper T cells (Tfh) were identified in C12, as marked by robust expression of CCR7, CXCR5, and ICOS. C15 and C20 were both defined as circulating regulatory T cells (Tregs), based on high expression of CD25 and low expression of CD127. We further defined C20 as activated Tregs based on increased expression of CTLA-4, ICOS, and CD38. Finally, we identified both CD8 + and CD4 + proliferating T cells in clusters C2 and C19, respectively, as defined by Ki-67 expression.

Baseline frequencies of T cell subsets were heterogeneous across different response groups (Fig. 2C, D). The patient with PD had low levels of both CD8 + (C3; *p* = 0.04 PD vs. SD) and CD4 + (C4; *p* = 0.09 PD vs. PR) naïve T cells and had the highest frequency of TfH (C12; 8.47% PD vs. 2.6% mean in CR/PR/SD). While no significant differences in the frequencies of Tregs (C15) or activated Tregs (C20) among total CD4 + T cells were observed, we did find that the percentage of activated Tregs within total Tregs was significantly higher in patients with SD (*p* = 0.01 SD vs. PR) (Fig. 2E). The fold change in the percentage of activated Tregs increased modestly in 5/8 patients from baseline to C2D1 (Fig. 2F) and returned to similar frequencies as baseline by post Cycle 3 (Fig. 2G). No significant differences in the change of Treg activation status over therapy were observed between patient response subgroups.

We next asked how T cell composition was altered over the course of therapy. Interpatient variability in frequency changes was high across all T cell subsets, with no significant differences between pre-treatment frequencies and frequencies at C2D1 or Post Cycle 3 (Supplemental Fig. 3A and B). Similarly, fold changes in T cell populations were heterogenous across patient response status (Supplemental Fig. 3C and 3D). Surprisingly, the cell populations that showed the greatest change over the course of therapy were an increase in naïve CD8 + and naïve CD4 + T cells from pre-treatment to C2D1 (*p* = 0.15 and *p* = 0.2). This increase was most dramatic in the patient with PD, with a fivefold increase in naïve CD8s and a twofold increase in naïve CD4s. Of interest, we also observed the PD patient to have a dramatic reduction from pre-treatment to C2D1 in CD8 + CD38 + EMRA T cells (C5, C8, C17), proliferating CD8 + and CD4 + T cells (C2 and C19), CD4 + effector cells (C13), and Tfh (C12). Overall, changes in T cell composition over the course of therapy were more modest for the patients with SD and PR, although we note both patients with SD demonstrated an increase in CD4 + proliferating T cells (C19, mea*n* = 2.5-fold change) from pre-treatment to C2D1. In contrast to the patient with PD, the patient with CR demonstrated unique changes in T cell composition from pre-treatment to C2D1. These included an increase in CD8 + CD57 + CD38 + EMRA T cells (C17, 1.5-fold change) and most notably an increase in CD8 + proliferating T cells (C2, 1.7-fold change) (Supplemental Fig. 3C).

**Fig. 3:**
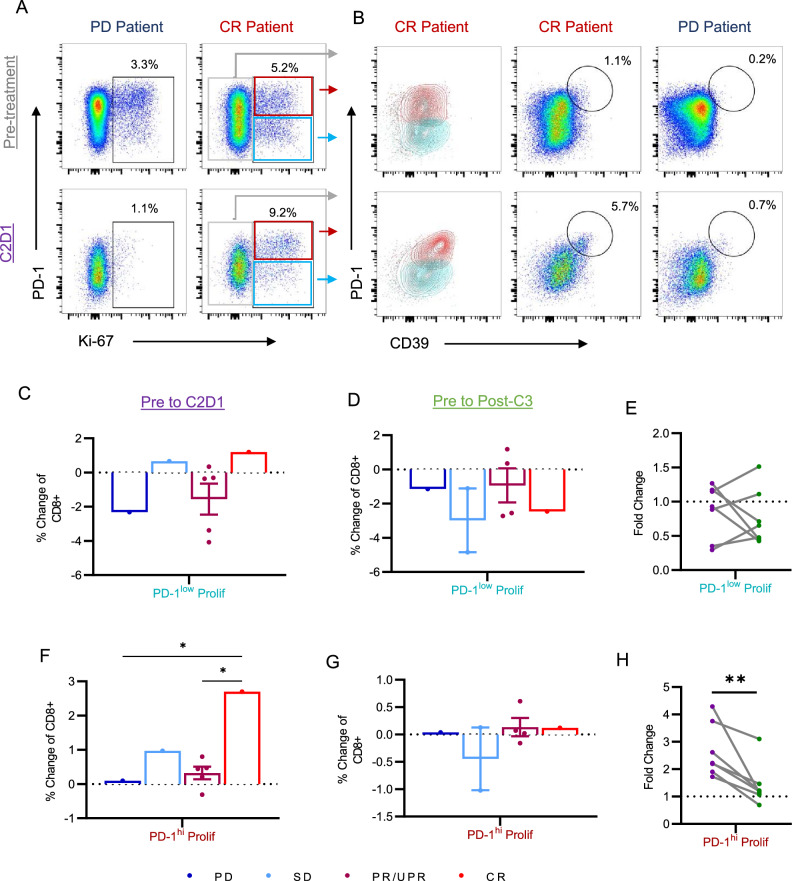
Dynamics of a proliferating CD8 + T cell population in patients treated with doxorubicin and pembrolizumab. Ki-67 + proliferating T cells were identified as shown in representative dot plots from pre-treatment and C2D1 (**A**). Frequencies indicate percentages among non-naïve CD8 + T cells. We further identified PD-1^hi^ (red box) and PD-1^lo^ (blue box) populations among proliferating Ki-67 + CD8 + T cells. Representative dot plots of PD-1 and CD39 expression are shown with frequencies indicating the percentage of PD-1^hi^ CD39 + T cells among non-naïve CD8 + T cells (**B**). Percentage changes from pre-treatment to C2D1 (**C**, **F)** and pre-treatment to post Cycle 3 (**D**, **G**) are shown for PD-1^lo^ Prolif (**C**, **F**) and PD-1^hi^ Prolif (**D**, **G**) populations. Fold change of PD-1^lo^ Prolif (**E**) and PD-1.^hi^ Prolif (**H**) populations with lines connecting matched patient fold changes from pre-treatment to C2D1 (purple dots) and pre-treatment to post Cycle 3 (green dots). **p < 0.01

### Expansion of a proliferative, exhausted CD8 + T cell population over the course of treatment

Among all patients, the increase in CD8 + proliferating T cells was greatest in the patient with CR (1.7-fold change vs. mean 0.8 in PD/SD/PR), which led us to further investigate this T cell subset. From pre-treatment to C2D1, CD8 + proliferating T cells increased from 5.2 to 9.2% in the patient with CR but reduced from 3.3 to 1.1% in the patient with PD (Fig. 3A). PD-1 expression was non-uniform among proliferating Ki-67 + T cells, yielding two sub-populations of proliferating CD8 + T cells (c2): PD-1^hi^ Prolif and PD-1^lo^ Prolif. We also observed that PD-1^hi^ Prolif increased expression of both PD-1 and CD39 from pre-treatment to C2D1 (Fig. 3B), yielding a PD-1^hi^ CD39 + phenotype associated with T cell exhaustion and tumor specificity [22, 23].

We next assessed dynamics of PD-1^hi^ Prolif and PD-1^lo^ Prolif CD8 + T cells over the course of therapy. At baseline, no significant differences were seen between patients in the frequencies of either population, although the patient with PD had the lowest frequency of PD-1^hi^ Prolif cells and one of the lowest frequencies of PD-1^lo^ Prolif cells (Supplemental Fig. 4A). From pre-treatment to C2D1, the PD-1^lo^ Prolif population decreased in *n* = 6/9 (67%) patients but demonstrated an increase of 1.2% in the patient with CR (Fig. 3C). In contrast, the PD-1^hi^ Prolif cell population increased in 8/9 patients from pre-treatment to C2D1 (Fig. 3F). This increase ranged from a modest expansion of 0.1% in the patient with PD to a significantly greater increase of 2.7% in the patient with CR (*p* = 0.01). We note that the increase in PD-1^hi^ Prolif cells in the patient with CR was an impressive 4.3-fold change, which was the highest fold change among all patients (mean 2.0 in PR/SD/PD) (Supplemental Fig. 4B). In 7/9 patients the frequency of PD-1^lo^ Prolif cells remained decreased compared to baseline by post Cycle 3 (Fig. 3D), with no significant differences observed in fold changes of this population from pre-treatment to C2D1 and post Cycle 3 (Fig. 3E). Notably, frequencies of PD-1^hi^ Prolif cells contracted significantly from C2D1 to post Cycle 3 (*p* = 0.005), with a return to near baseline frequencies in the majority of patients (Fig. 3G, H, Supplemental Fig. 4C). In the context of all identified T cell subsets, PD-1^hi^ Prolif cells demonstrated the greatest increase in fold change from pre-treatment to C2D1 (Supplemental Fig. 4D). In contrast, PD-1^lo^ Prolif CD8 + T cells demonstrated the greatest decrease in fold change from pre-treatment to C3D1 (Supplemental Fig. 4E).

**Fig. 4:**
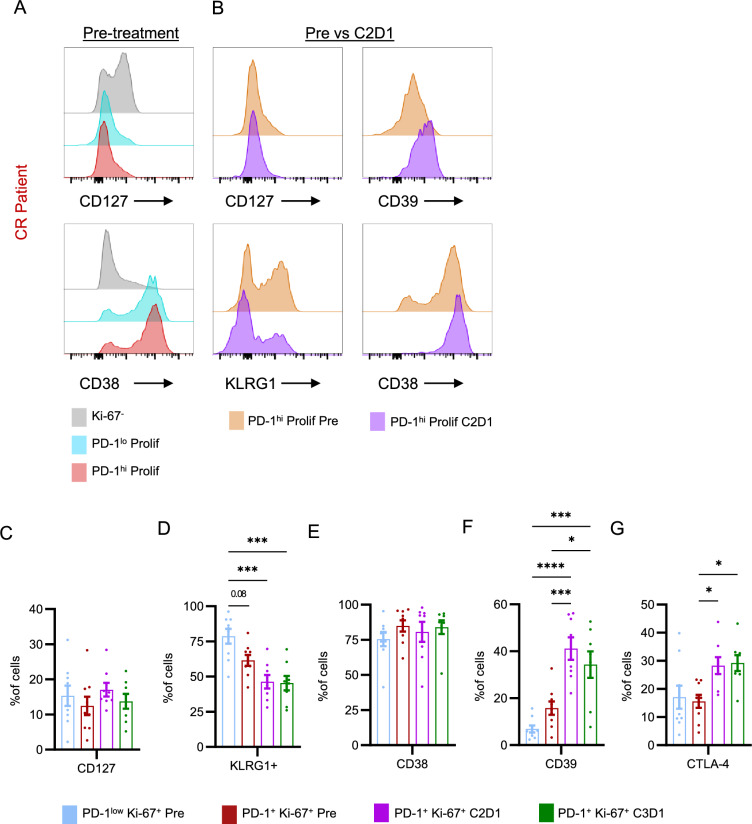
Phenotype changes of PD-1 high proliferating T cells over the course of treatment with doxorubicin and pembrolizumab for one patient with CR. CD127 expression and CD38 expression was compared between Ki-67- (gray), PD-1^lo^ Prolif (blue) and PD-1^hi^ Prolif (red) T cells at pre-treatment (**A**). PD-1^lo^ Prolif T cells at pre-treatment (orange) were also compared to PD-1.^hi^ Prolif T cells at C2D1 (purple) for expression of CD127, CD39, KLRG1, and CD38. Bar graphs depicting percent of CD8 + T cell subsets are shown for CD127 (**C**), KLRG1 (**D**), CD38 (**E**), CD39 (**F**), and CTLA-4 (**G**). *p < 0.05; ***p < 0.001

Since the generation of a PD-1^hi^ CD39 + phenotype was a clear outcome of combined doxorubicin and pembrolizumab treatment, we next set to further evaluate characteristics of this T cell phenotype. Co-expression of high levels of PD-1 and CD39 has been described by us and others to mark CD8 + T cells with an exhausted T cell phenotype [24, 25]. As compared to Ki-67- CD8 + T cells, we observed pre-treatment PD-1^lo^ Prolif and PD-1^hi^ Prolif to express reduced levels of CD127 and increased levels of CD38 (Fig. 4A). This suggested that prior to exposure to pembrolizumab both Ki-67 + CD8 + T cell populations were already in a highly differentiated and activated state. From pre-treatment to C2D1, PD-1^hi^ Prolif cells maintained low levels of CD127, but also lost expression of KLRG1 (Fig. 4B–D), resulting in a CD127- KLRG1- phenotype associated with terminal differentiation. PD-1^hi^ Prolif cells also maintained high levels of CD38 and gained expression of CD39 (Fig. 4B–F). Finally, we observed increased expression of CTLA-4 on PD-1^hi^ Prolif cells over the course of treatment (Fig. 4G), which also has been described to be upregulated on CD8 + exhausted T cells.

## Discussion

Although limited by small sample size, the results of the current trial provide evidence that doxorubicin can be safely combined with pembrolizumab with *n* = 6/9 (67%) ORR in patients with mTNBC who were not pre-selected for PD-L1. Immune toxicities were consistent with known pembrolizumab toxicity as listed in package insert. The most significant grade ≥ 3 irAE was neutropenia which occurred in *n* = 4/10 (40%) of patients. The KEYNOTE-355 trial already established pembrolizumab plus chemotherapy for standard management of first-line patients with PD-L1 + TNBC (≥ 10% 22C3 Ventana) [8]. The data presented by this study may provide proof of concept for the utility of anthracycline plus pembrolizumab combination for treatment of mTNBC.

Several studies have demonstrated utility of chemo-immunotherapy combination in treatment of mTNBC. FDA accelerated approval was granted to atezolizumab in March 2019 based on data from IMpassion130 trial (NCT02425891) which demonstrated a statistically significant benefit to PFS with the atezolizumab and nab-paclitaxel vs. nab-paclitaxel alone (HR, 0.60; 95% CI 0.48–0.77; *P* < 0.0001) [4]. Continued approval of atezolizumab was contingent upon results of the IMpassion131 trial (NCT03125902), which failed to meet the primary end point of PFS benefit as first-line treatment of patients with PD-L1 + mTNBC using the SP142 antibody (HR, 0.82; 95% CI 0.60–1.12; *P* = 0.20). Additionally, there was no difference in OS in PD-L1 + mTNBC (HR 1.11, 95% CI 0.76–1.64) nor the intention to treat (ITT) population, which led to the withdrawal of FDA approval in August 2021. In the KEYNOTE-355 study, first-line patients with PD-L1 + TNBC defined by a CPS ≥ 10 pembrolizumab plus chemotherapy had improved PFS compared with chemotherapy alone (9.7 vs. 5.6 months; HR 0.65, 95% CI 0.49–0.86) [8, 10]. In the ENHANCE1 trial, eribulin in combination with pembrolizumab achieved an ORR of 26% in the first-line setting, and 22% in the 2–3 lines setting. In selected patients with PD-L1 + disease, an ORR of 34.5% and 24.4% was identified for first-line and 2–3-line patients, respectively. Shah et al. reported an ORR of 26% and CBR of 28% (*n* = 15) in a cohort of TNBC treated with capecitabine and pembrolizumab combination [26]. Page et al. reported capecitabine and pembrolizumab demonstrated a 12-week ORR of 43% and PFS of 5.6 months [27]. The higher response rate may be related to its use in earlier lines of therapy (first line in 79% of patients. An earlier dataset demonstrated an ORR of 11% or 31% with single agent doxorubicin or liposomal doxorubicin in patient with metastatic breast cancer. In the Intergroup E1193 trial, first-line doxorubicin had an ORR of 36% in MBC [28, 29]. It has been well documented in KEYNOTE-086 and KEYNOTE-119 that the single agent pembrolizumab has a limited ORR of 5–12% in ≥ 2-line setting [30, 31]. In the TONIC trial, the immune modulatory activity of 15 mg IV weekly × 2 doxorubicin (*n* = 17) was demonstrated using sequential treatment of anthracycline and nivolumab (3 mg/kg every 2 weeks for 3 cycles), resulted in ORR of 35% in comparison with PD-L1 blockade (nivolumab) alone (*n* = 12) with ORR of 17%. Immune-related genes that were upregulated in doxorubicin-treated patients included inflammation, JAK-STAT, and TNF-alpha signaling [32]. In a breast cancer mouse model, doxorubicin selectively depleted myeloid-derived suppressor cells (MDSC) from the tumor microenvironment [15]. In addition, immune checkpoint blockade improved chemotherapy in the PyMT mammary carcinoma mouse model” [33]. In our study, an ORR of 67% in response-evaluable patients with doxorubicin and pembrolizumab in anthracycline-naïve PD-L1 unselected patients is encouraging, while we acknowledge the limitation in our sample size. Our data may provide additional options for patients who have not previously received anthracycline.

Anthracyclines are among the most active agents for treatment of breast cancer. Anthracycline elicited immunogenic apoptosis in the preclinical setting [14]. There are preclinical data that suggests doxorubicin downregulates B7-H1 (PD-L1) expression [34]. Furthermore, Alizadeh et al. reported potential immune modulatory effects of anthracycline by demonstrating that doxorubicin eliminated myeloid-derived suppressor cells and increased CD4 + and CD8 + T cells using a breast cancer mouse model [15, 34, 35]. Doxorubicin has been associated with myeloid-derived suppressor cell (MDSC) depletion [15], an increase in the level of type I interferons [36] and induction of immunogenic cell death [14]. The combination of pembrolizumab and anthracycline was tested in sarcoma in a phase I trial, and the regimen is well tolerated with modest efficacy. Here, doxorubicin and pembrolizumab showed promising activity in anthracycline-naïve mTNBC in the limited number of patients treated. Other limitations of this study include the nonrandomized, single-arm trial nature; the molecularly heterogenous population (including different lines of therapy); and tumor PD-L1 expression status. Of 8 patients who had PD-L1 tested, 4 were PD-L1 positive and 4 were PD-L1 negative. The clinical expansion of these results is challenging since most patients with TNBC have previously received an anthracycline-containing regimen in the neoadjuvant or adjuvant setting. This resulted in the accrual limitations that resulted in early termination of this study. Future clinical trials with a larger patient cohort and a randomized design are required to evaluate this combination and explore potential predictors of response to better identify the subset of patients who may benefit from this chemo-immunotherapy combination.

Our profiling of T cell dynamics in mTNBC patients treated with doxorubicin and pembrolizumab provides insight into immunotherapy response mechanisms. In this study, baseline immune biomarkers were not associated with response to treatment. In the patient with PD, significantly low levels of naïve CD8 + and CD4 + T cells at baseline indicating the presence of naïve CD8 + and CD4 + T cell is necessary for response to immune checkpoint blockade. Indeed, recent evidence has suggested that PD-1 blockade instills anti-tumor T cell immunity via the generation of non-preexisting T cell clonotypes [37]. Our data also points to a robust expansion of a proliferating subset of CD8 + T cells within one cycle of pembrolizumab treatment. These proliferating CD8 + T cells were phenotyped as highly activated, with increasing expression of PD-1, CD39, CD38, and CTLA-4 and acquisition of an exhausted T cell phenotype over the course of therapy. Similar findings were previously identified in non-small cell lung cancer patients treated with pembrolizumab [38], demonstrating the ability for PD-1 blockade to stimulate peripheral T cell expansion and activation. In parallel, tumor infiltrating exhausted T cells and exhausted-like T cells have been associated with improved TNBC patient survival and response to immunotherapy in estrogen receptor-positive breast cancer [39, 40]. Further studies clarifying what preexisting features of either peripheral or tumor infiltrating T cells are needed for clinical response to PD-1 blockade will enable better selection of patients for treatment with immunotherapy.

Importantly, we find that the expansion and not baseline percentages of proliferative exhausted CD8 + T cells that correlates with response to PD-1 blockade, which is in agreement with studies of pembrolizumab-treated melanoma patients [41]. We also show that the expanded exhausted CD8 + T cell population largely collapses by post Cycle 3, perhaps suggesting a lack of benefit to continued PD-1 blockade. Indeed, others have shown that a single dose of anti-PD-1 therapy could amplify meaningful anti-tumor CD8 + T cell responses in the neoadjuvant setting [42]. Long-term studies of the peripheral T cell response in ICI-treated mTNBC patients are critically needed to understand mechanisms of durable tumor-specific T cell immunity.

Increasing attention is now being turned toward understanding how existing cytotoxic chemotherapies generate or shape an immune response, and how these may best partner with immunotherapies [43]. In support of our findings, a separate study found that mTNBC patients treated with doxorubicin and pembrolizumab were more likely than patients treated with capecitabine or paclitaxel to expand new T cell clones over the course of therapy [44]. Recently, another study found that pre-treatment levels of tumor infiltrating CXCL13 + exhausted CD8 + T cells were predictive of response to paclitaxel combined with atezolizumab in TNBC patients [45]. Critically, the authors also found that paclitaxel limited the expansion of anti-tumor immune cells driven by atezolizumab treatment, highlighting the importance of improved treatment paradigms of paired chemotherapies and immunotherapies. Thus, cytotoxic chemotherapies may yield transient lymphodepletion, reduction of immunosuppressive cell types, and enhanced tumor immunogenicity that profoundly alter immune cell dynamics over the course of immunotherapy [46, 47]. Novel clinical trial designs with improved chemotherapy dosing strategies and immunotherapy partners are needed to fully harness immune-based treatments of mTNBC.

In conclusion, anthracycline-naïve patients with mTNBC treated with the combination of pembrolizumab and doxorubicin showed an encouraging response rate and robust T cell responses. The combination was generally well tolerated, and the utility of this combination needs to be further studied.

### Supplementary Information

Below is the link to the electronic supplementary material.

Supplemental Fig. 1. Broad immune characterization of peripheral blood of patients treated with doxorubicin and pembrolizumab at baseline. A) Pre-treatment peripheral blood samples were examined by flow cytometry for lymphocyte composition frequencies, including CD3 + T cells, CD3 − CD56 + natural killer (NK) cells, CD3 + CD56 + natural killer T (NKT) cells, TCRγδ T cells, and CD19 + B cells. B) The composition of NK cells was further assessed for CD56bright (early), CD16bright (mature), and CD56dim CD16dim (terminal) NK cell subsets. C) B cell subsets were assessed as IgD + CD27- (Naïve), IgD- CD27 + (memory), or CD38 + plasmablasts. D) Monocyte subsets were assessed as a fraction of a myeloid cell gate for frequencies of CD14 + CD16- classical monocytes (clMono), CD14 + CD16 + intermediate monocytes (intMono), and CD14- CD16 + non-classical monocytes (ncMono). E) Dendritic cell subsets were similarly assessed as a fraction of a myeloid cell gate for frequencies of CD141 + dendritic cells (cDC1), CD1c + dendritic cells (cDC2), and CD123 + plasmacytoid dendritic cells (pDC). F) Percent change from pre-treatment to C2D1 (purple) and from pre-treatment to post Cycle 3 (green) is shown. Patient with disease progression (PD, n = 1) is depicted in dark blue, stable disease (SD, n = 2) in light blue, confirmed and unconfirmed partial response (PR = 5) in dark red, and complete response (CR, n = 1) in light red. *p < 0.05. Supplemental Fig. 2. Canonical T cell subsets at baseline. CD8 + T cells (A) and CD4 + T cells (B) were further assessed by flow cytometry for frequencies of naïve (CCR7 + , CD45RA +), central memory (CM; CCR7 + , CD45RA-), effector memory (EM; CCR7-, CD45RA-), and effector memory CD45RA + (EMRA, CCR7-, CD45RA +) cell subsets. Frequencies of regulatory T cells (Tregs) were also assessed within CD4 + T cells. Data are shown separately for patients with PD (dark blue), SD (light blue), PR (dark red), and CR (light red). Supplemental Fig. 3. Detailed T cell composition changes over the course of therapy in patients treated with doxorubicin and pembrolizumab. Percentage change of all identified CD8 + T cell subsets (A) and CD4 + T cell subsets (B) from pre-treatment to C2D1 (purple) and pre-treatment to post Cycle 3 (red). Fold change in CD8 + (C) and CD4 + (D) T cell subsets are shown separately for patients with PD (dark blue), SD (light blue), PR (dark red), and CR (light red). Supplemental Fig. 4. Detailed dynamics of proliferating CD8 + T cells in patients treated with doxorubicin plus pembrolizumab. Pre-treatment frequencies (A), fold change from pre-treatment to C2D1 (B), and fold change from pre-treatment to C3D1 (C) are shown for PD-1low Ki-67 + and PD-1high Ki-67 + CD8 + T cells. PD (dark red), SD (light red), PR (light blue), and CR (dark blue). Fold change of all T cell subsets, with the inclusion of identified PD-1low Ki-67 + and PD-1high Ki-67 + populations, from pre-treatment to C2D1 (D) and from pre-treatment to C3D1 (E). (PDF 243 kb)

## Data Availability

All data and materials are presented in the article and additional files. Raw data are available upon request.

## Abbreviations

AE: Adverse events
CBR: Clinical benefit rate
C2D1: Cycle 2 day 1
C3D1: Cycle 3 day 1
CPS: Combined positive score
CR: Complete response
DOR: Duration of response
ER: Estrogen receptor
FDA: Food and Drug Administration
FFPE: Formalin-fixed paraffin-embedded
HER2: Human epidermal growth factor receptor 2
H&E: Hematoxylin and eosin
ICI: Immune checkpoint inhibitor
IHC: Immunohistochemistry
irAEs: Immune-related adverse events
IRB: Institutional review board
ITT: Intention to treat
mTNBC: Metastatic triple-negative breast cancer
ORR: Objective response rate
OS: Overall survival
PD-L1 +: PD-L1–positive
PFS: Progression-free survival
PR: Progression receptor
PR: Partial response
RP2D: Recommended phase II dose
SD: Stable disease
sTIL: Stromal TIL
TNBC: Triple-negative breast cancer

## References

[1] Dent R, Trudeau M, Pritchard KI, Hanna WM, Kahn HK, Sawka CA (2007). Triple-negative breast cancer: clinical features and patterns of recurrence. Clin Cancer Res.

[2] Litton JK, Rugo HS, Ettl J, Hurvitz SA, Gonçalves A, Lee KH (2018). Talazoparib in patients with advanced breast cancer and a germline BRCA mutation. N Engl J Med.

[3] Robson M, Im SA, Senkus E, Xu B, Domchek SM, Masuda N (2017). Olaparib for metastatic breast cancer in patients with a germline BRCA mutation. N Engl J Med.

[4] Schmid P, Rugo HS, Adams S, Schneeweiss A, Barrios CH, Iwata H (2020). Atezolizumab plus nab-paclitaxel as first-line treatment for unresectable, locally advanced or metastatic triple-negative breast cancer (IMpassion130): updated efficacy results from a randomised, double-blind, placebo-controlled, phase 3 trial. Lancet Oncol.

[5] Bardia A, Mayer IA, Vahdat LT, Tolaney SM, Isakoff SJ, Diamond JR (2019). Sacituzumab govitecan-hziy in refractory metastatic triple-negative breast cancer. N Engl J Med.

[6] Li CH, Karantza V, Aktan G, Lala M (2019). Current treatment landscape for patients with locally recurrent inoperable or metastatic triple-negative breast cancer: a systematic literature review. Breast Cancer Res.

[7] Schmid P, Adams S, Rugo HS, Schneeweiss A, Barrios CH, Iwata H (2018). Atezolizumab and nab-paclitaxel in advanced triple-negative breast cancer. N Engl J Med.

[8] Cortes J, Cescon DW, Rugo HS, Nowecki Z, Im SA, Yusof MM (2020). Pembrolizumab plus chemotherapy versus placebo plus chemotherapy for previously untreated locally recurrent inoperable or metastatic triple-negative breast cancer (KEYNOTE-355): a randomised, placebo-controlled, double-blind, phase 3 clinical trial. Lancet.

[9] Keytruda (pembrolizumab) [package insert] (2020) Whitehouse station, Merck Sharp & Dohme Corp, NJ

[10] Tolaney SM, Kalinsky K, Kaklamani VG, D'Adamo DR, Aktan G, Tsai ML (2021). Eribulin plus pembrolizumab in patients with metastatic triple-negative breast cancer (ENHANCE 1): a phase Ib/II study. Clin Cancer Res.

[11] Pandy JGP, Balolong-Garcia JC, Cruz-Ordinario MVB, Que FVF (2019). Triple negative breast cancer and platinum-based systemic treatment: a meta-analysis and systematic review. BMC Cancer.

[12] Yuan Y, Lee JS, Yost SE, Li SM, Frankel PH, Ruel C (2021). Phase II trial of neoadjuvant carboplatin and nab-paclitaxel in patients with triple-negative breast cancer. Oncologist.

[13] van der Zanden SY, Qiao X, Neefjes J (2021). New insights into the activities and toxicities of the old anticancer drug doxorubicin. Febs J.

[14] Casares N, Pequignot MO, Tesniere A, Ghiringhelli F, Roux S, Chaput N (2005). Caspase-dependent immunogenicity of doxorubicin-induced tumor cell death. J Exp Med.

[15] Alizadeh D, Trad M, Hanke NT, Larmonier CB, Janikashvili N, Bonnotte B (2014). Doxorubicin eliminates myeloid-derived suppressor cells and enhances the efficacy of adoptive T-cell transfer in breast cancer. Cancer Res.

[16] Mattarollo SR, Loi S, Duret H, Ma Y, Zitvogel L, Smyth MJ (2011). Pivotal role of innate and adaptive immunity in anthracycline chemotherapy of established tumors. Cancer Res.

[17] Frankel PH, Chung V, Tuscano J, Siddiqi T, Sampath S, Longmate J (2020). Model of a queuing approach for patient accrual in phase 1 oncology studies. JAMA Netw Open.

[18] Salgado R, Denkert C, Demaria S, Sirtaine N, Klauschen F, Pruneri G (2015). The evaluation of tumor-infiltrating lymphocytes (TILs) in breast cancer: recommendations by an International TILs Working Group 2014. Ann Oncol.

[19] Muro K, Chung HC, Shankaran V, Geva R, Catenacci D, Gupta S (2016). Pembrolizumab for patients with PD-L1-positive advanced gastric cancer (KEYNOTE-012): a multicentre, open-label, phase 1b trial. Lancet Oncol.

[20] Dolled-Filhart M, Locke D, Murphy T, Lynch F, Yearley JH, Frisman D (2016). Development of a prototype immunohistochemistry assay to measure programmed death ligand-1 expression in tumor tissue. Arch Pathol Lab Med.

[21] Chevrier S, Crowell HL, Zanotelli VRT, Engler S, Robinson MD, Bodenmiller B (2018). Compensation of signal spillover in suspension and imaging mass cytometry. Cell Syst.

[22] Gros A, Robbins PF, Yao X, Li YF, Turcotte S, Tran E (2014). PD-1 identifies the patient-specific CD8^+^ tumor-reactive repertoire infiltrating human tumors. J Clin Invest.

[23] Simoni Y, Becht E, Fehlings M, Loh CY, Koo SL, Teng KWW (2018). Bystander CD8(+) T cells are abundant and phenotypically distinct in human tumour infiltrates. Nature.

[24] Egelston CA, Guo W, Tan J, Avalos C, Simons DL, Lim MH (2022). Tumor-infiltrating exhausted CD8+ T cells dictate reduced survival in premenopausal estrogen receptor-positive breast cancer. JCI Insight.

[25] Canale FP, Ramello MC, Núñez N, Araujo Furlan CL, Bossio SN, Gorosito Serrán M (2018). CD39 expression defines cell exhaustion in tumor-infiltrating CD8(+) T cells. Cancer Res.

[26] Shah AN, Flaum L, Helenowski I, Santa-Maria CA, Jain S, Rademaker A (2020). Phase II study of pembrolizumab and capecitabine for triple negative and hormone receptor-positive, HER2-negative endocrine-refractory metastatic breast cancer. J Immunother Cancer.

[27] Page DB, Chun B, Pucilowska J, Kim I, Sanchez K, Redmond WL (2019). Pembrolizumab (pembro) with paclitaxel (taxol) or capecitabine (cape) as early treatment of metastatic triple-negative breast cancer (mTNBC). J Clin Oncol.

[28] Gennari A, D'Amico M (2011). Anthracyclines in the management of metastatic breast cancer: state of the art. Eur J Cancer Suppl.

[29] Sledge GW, Neuberg D, Bernardo P, Ingle JN, Martino S, Rowinsky EK (2003). Phase III trial of doxorubicin, paclitaxel, and the combination of doxorubicin and paclitaxel as front-line chemotherapy for metastatic breast cancer: an intergroup trial (E1193). J Clin Oncol.

[30] Adams S, Loi S, Toppmeyer D, Cescon DW, De Laurentiis M, Nanda R (2019). Pembrolizumab monotherapy for previously untreated, PD-L1-positive, metastatic triple-negative breast cancer: cohort B of the phase II KEYNOTE-086 study. Ann Oncol.

[31] Cortés J, Lipatov O, Im SA, Gonçalves A, Lee KS, Schmid P (2019). LBA21 - KEYNOTE-119: Phase III study of pembrolizumab (pembro) versus single-agent chemotherapy (chemo) for metastatic triple negative breast cancer (mTNBC). Ann Oncol.

[32] Voorwerk L, Slagter M, Horlings HM, Sikorska K, van de Vijver KK, de Maaker M (2019). Immune induction strategies in metastatic triple-negative breast cancer to enhance the sensitivity to PD-1 blockade: the TONIC trial. Nat Med.

[33] Sirait-Fischer E, Olesch C, Fink AF, Berkefeld M, Huard A, Schmid T (2020). Immune checkpoint blockade improves chemotherapy in the pymt mammary carcinoma mouse model. Front Oncol.

[34] Ghebeh H, Lehe C, Barhoush E, Al-Romaih K, Tulbah A, Al-Alwan M (2010). Doxorubicin downregulates cell surface B7–H1 expression and upregulates its nuclear expression in breast cancer cells: role of B7–H1 as an anti-apoptotic molecule. Breast Cancer Res.

[35] Pollack SM, Redman MW, Baker KK, Wagner MJ, Schroeder BA, Loggers ET (2020). Assessment of doxorubicin and pembrolizumab in patients with advanced anthracycline-naive sarcoma: a phase 1/2 nonrandomized clinical trial. JAMA Oncol.

[36] Sistigu A, Yamazaki T, Vacchelli E, Chaba K, Enot DP, Adam J (2014). Cancer cell-autonomous contribution of type I interferon signaling to the efficacy of chemotherapy. Nat Med.

[37] Yost KE, Satpathy AT, Wells DK, Qi Y, Wang C, Kageyama R (2019). Clonal replacement of tumor-specific T cells following PD-1 blockade. Nat Med.

[38] Kamphorst AO, Pillai RN, Yang S, Nasti TH, Akondy RS, Wieland A (2017). Proliferation of PD-1+ CD8 T cells in peripheral blood after PD-1-targeted therapy in lung cancer patients. Proc Natl Acad Sci U S A.

[39] Savas P, Virassamy B, Ye C, Salim A, Mintoff CP, Caramia F (2018). Single-cell profiling of breast cancer T cells reveals a tissue-resident memory subset associated with improved prognosis. Nat Med.

[40] Terranova-Barberio M, Pawlowska N, Dhawan M, Moasser M, Chien AJ, Melisko ME (2020). Exhausted T cell signature predicts immunotherapy response in ER-positive breast cancer. Nat Commun.

[41] Huang AC, Postow MA, Orlowski RJ, Mick R, Bengsch B, Manne S (2017). T-cell invigoration to tumour burden ratio associated with anti-PD-1 response. Nature.

[42] Huang AC, Orlowski RJ, Xu X, Mick R, George SM, Yan PK (2019). A single dose of neoadjuvant PD-1 blockade predicts clinical outcomes in resectable melanoma. Nat Med.

[43] Salas-Benito D, Pérez-Gracia JL, Ponz-Sarvisé M, Rodriguez-Ruiz ME, Martínez-Forero I, Castañón E (2021). Paradigms on immunotherapy combinations with chemotherapy. Cancer Discov.

[44] Chun B, Pucilowska J, Chang S, Kim I, Nikitin B, Koguchi Y (2022). Changes in T-cell subsets and clonal repertoire during chemoimmunotherapy with pembrolizumab and paclitaxel or capecitabine for metastatic triple-negative breast cancer. J Immuno Ther Cancer.

[45] Zhang Y, Chen H, Mo H, Hu X, Gao R, Zhao Y (2021). Single-cell analyses reveal key immune cell subsets associated with response to PD-L1 blockade in triple-negative breast cancer. Cancer Cell.

[46] Emens LA, Middleton G (2015). The interplay of immunotherapy and chemotherapy: harnessing potential synergies. Cancer Immunol Res.

[47] Bracci L, Schiavoni G, Sistigu A, Belardelli F (2014). Immune-based mechanisms of cytotoxic chemotherapy: implications for the design of novel and rationale-based combined treatments against cancer. Cell Death Differ.

