# Supplementary figures and images for: Immunogenicity and efficacy of pembrolizumab and doxorubicin in a phase I trial for patients with metastatic triple-negative breast cancer
Source: Cancer Immunol Immunother. 2023 Jun 9;72(9):3013–27. doi: 10.1007/s00262-023-03470-y (PMC10412661; doi:10.1007/s00262-023-03470-y)

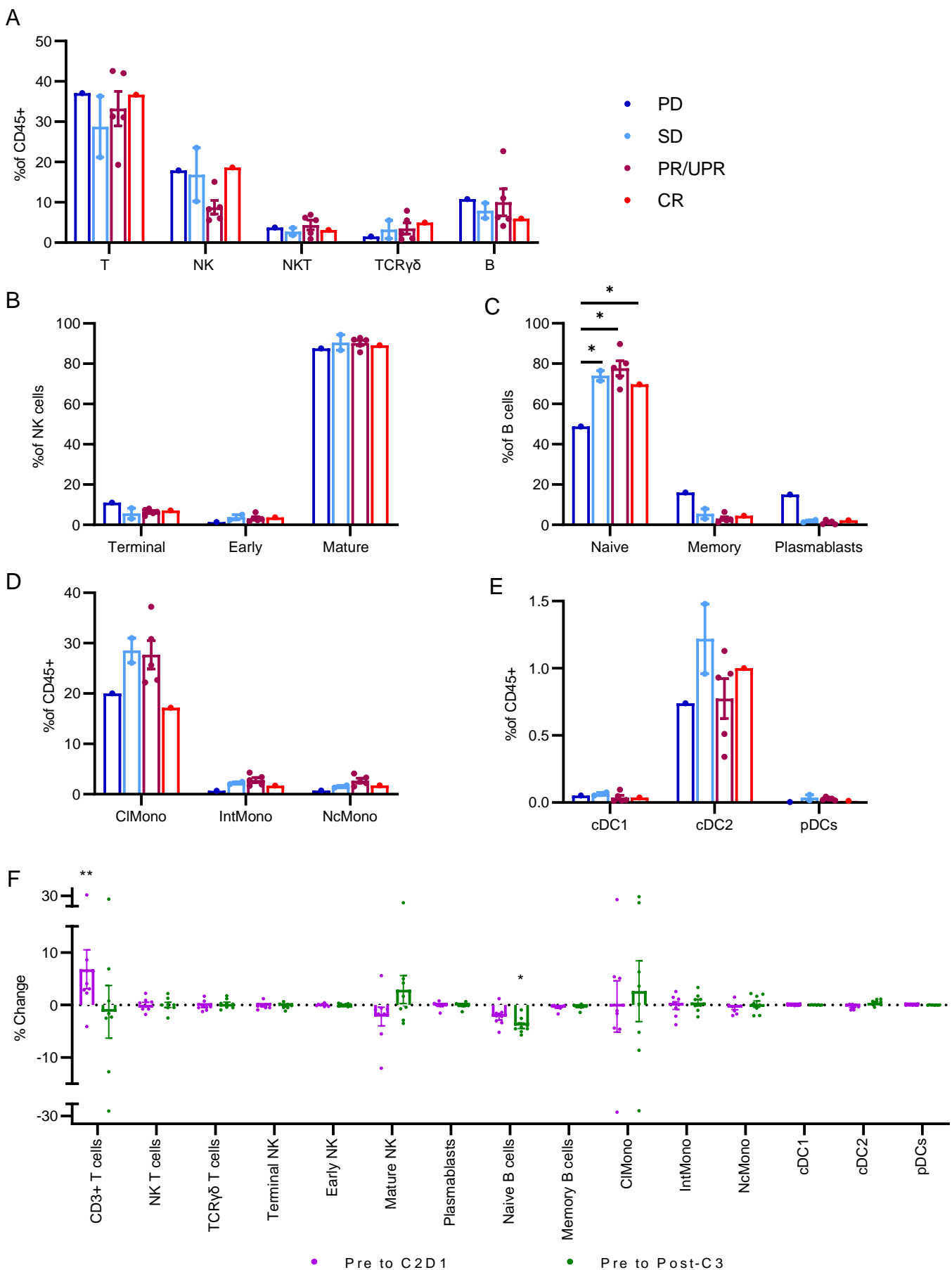

Supplemental Figure 1

A

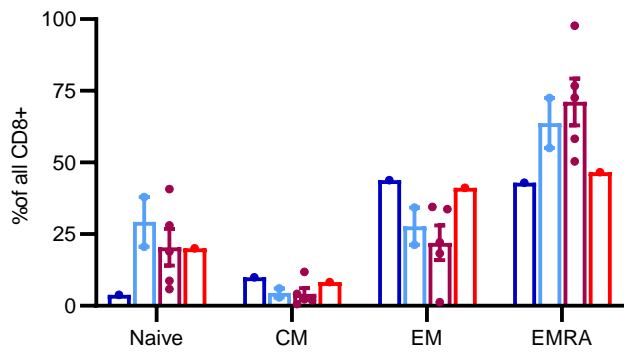

B

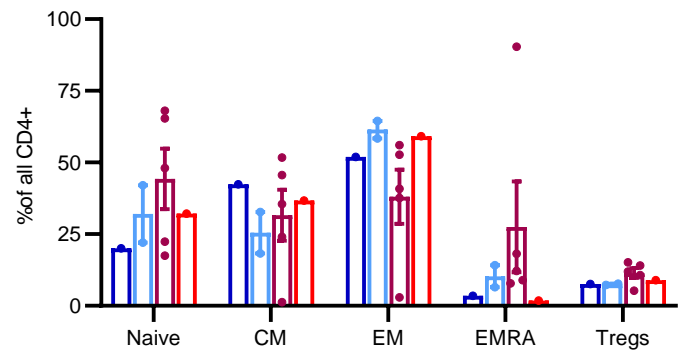

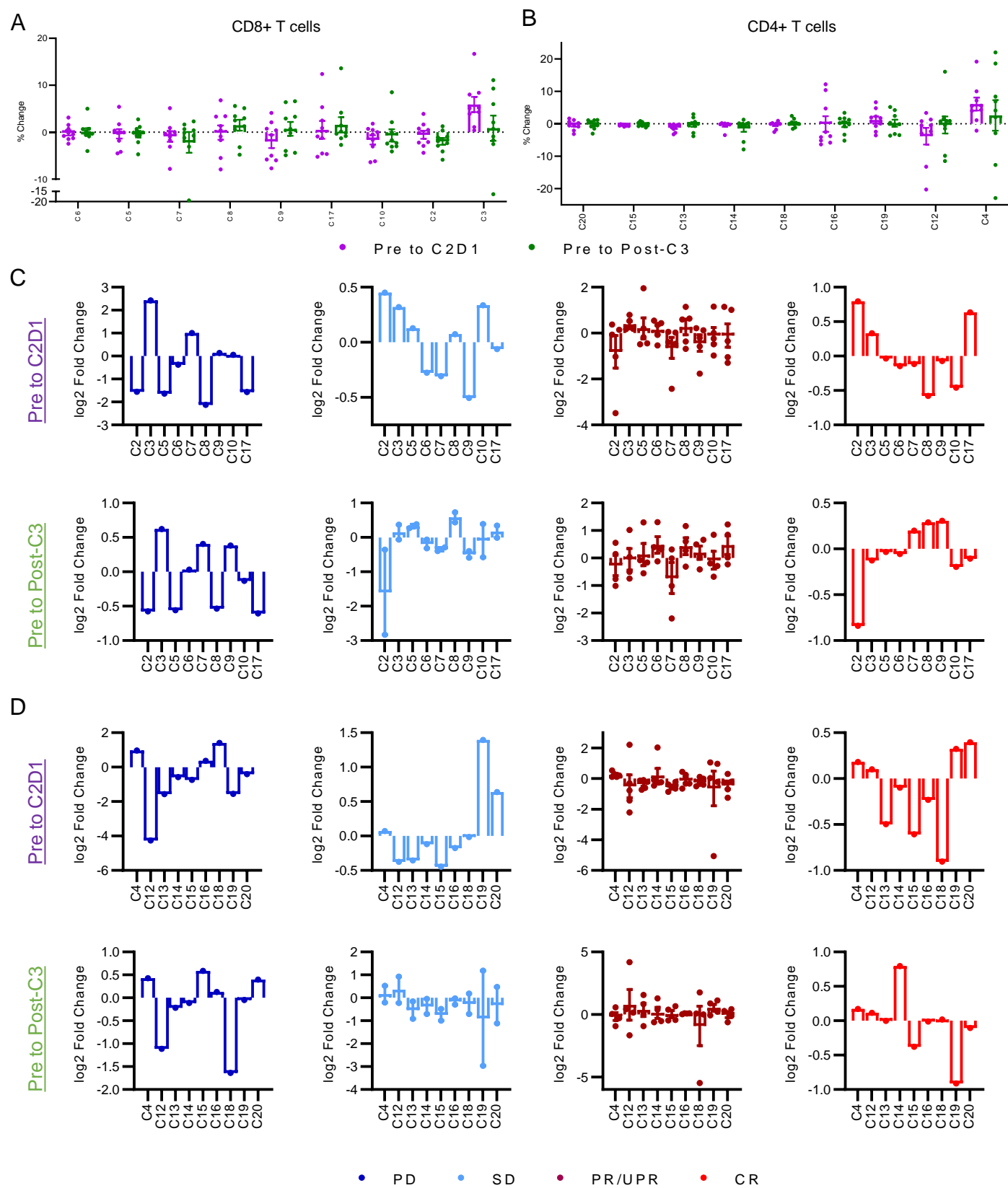

Supplemental Figure 3

A

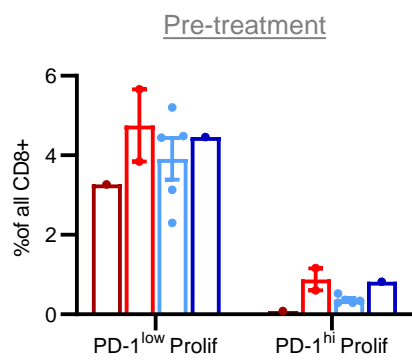

B

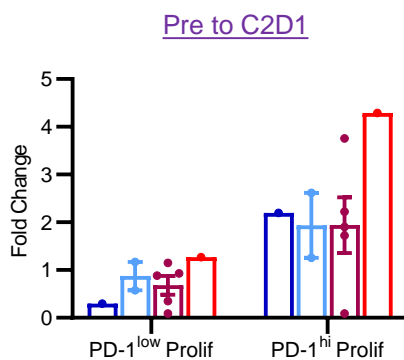

C

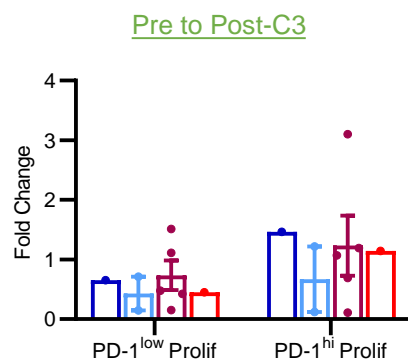

D

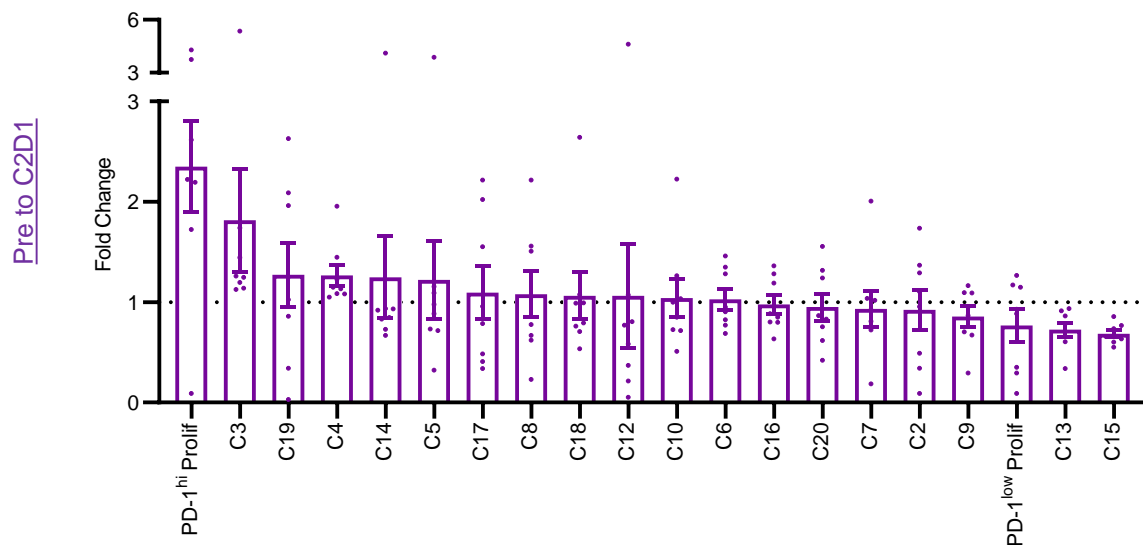

E

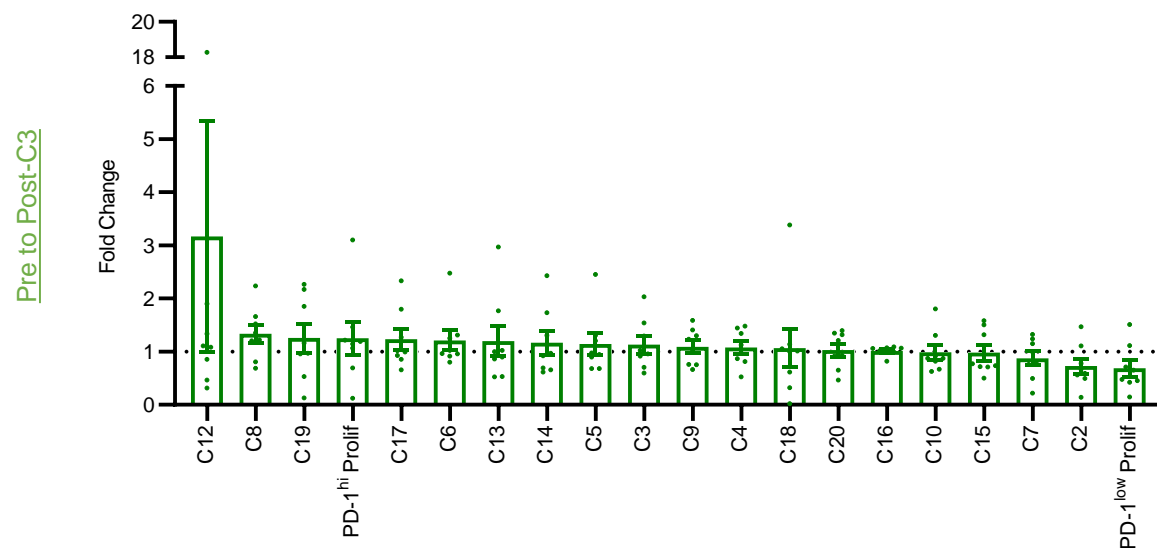

Supplement: Supplementary file 1 — Supplemental Fig. 1. Broad immune characterization of peripheral blood of patients treated with doxorubicin and pembrolizumab at baseline. A) Pre-treatment peripheral blood samples were examined by flow cytometry for lymphocyte composition frequencies, including CD3 + T cells, CD3 − CD56 + natural killer (NK) cells, CD3 + CD56 + natural killer T (NKT) cells, TCRγδ T cells, and CD19 + B cells. B) The composition of NK cells was further assessed for CD56bright (early), CD16bright (mature), and CD56dim CD16dim (terminal) NK cell subsets. C) B cell subsets were assessed as IgD + CD27- (Naïve), IgD- CD27 + (memory), or CD38 + plasmablasts. D) Monocyte subsets were assessed as a fraction of a myeloid cell gate for frequencies of CD14 + CD16- classical monocytes (clMono), CD14 + CD16 + intermediate monocytes (intMono), and CD14- CD16 + non-classical monocytes (ncMono). E) Dendritic cell subsets were similarly assessed as a fraction of a myeloid cell gate for frequencies of CD141 + dendritic cells (cDC1), CD1c + dendritic cells (cDC2), and CD123 + plasmacytoid dendritic cells (pDC). F) Percent change from pre-treatment to C2D1 (purple) and from pre-treatment to post Cycle 3 (green) is shown. Patient with disease progression (PD, n = 1) is depicted in dark blue, stable disease (SD, n = 2) in light blue, confirmed and unconfirmed partial response (PR = 5) in dark red, and complete response (CR, n = 1) in light red. *p < 0.05. Supplemental Fig. 2. Canonical T cell subsets at baseline. CD8 + T cells (A) and CD4 + T cells (B) were further assessed by flow cytometry for frequencies of naïve (CCR7 + , CD45RA +), central memory (CM; CCR7 + , CD45RA-), effector memory (EM; CCR7-, CD45RA-), and effector memory CD45RA + (EMRA, CCR7-, CD45RA +) cell subsets. Frequencies of regulatory T cells (Tregs) were also assessed within CD4 + T cells. Data are shown separately for patients with PD (dark blue), SD (light blue), PR (dark red), and CR (light red). Supplemental Fig. 3. Detailed T cell [file 262_2023_3470_MOESM1_ESM.pdf]
